# Bibliometric analysis of publication trends on ocular hygiene and infections in the past two decades

**DOI:** 10.3205/dgkh000489

**Published:** 2024-06-21

**Authors:** Mohsan Ali, Bisal Naseer, Rawal Alias Insaf Ahmed, Muhammad Talha, Moeez Saqib, Amar Anwar

**Affiliations:** 1 MBBS Graduate, King Edward Medical University, Lahore, Pakistan; 2 Provincial Disease Surveillance & Response Unit, Hyderabad, Sindh, Pakistan; 3 MBBS Scholar, Combined Military Hospital Medical College, Lahore, Pakistan

## Abstract

**Background:**

Ocular hygiene encompasses a spectrum of measures to initiate and maintain adequate ocular cleanliness to prevent eye infections and their further transmission. These infections affect all age groups and can lead to severe complications such as blindness. Nearly 1 billion cases could have been prevented out of over 2.2 billion people that are visually impaired worldwide. This bibliometric analysis focuses on the papers published on ocular hygiene and infections.

**Methods:**

We searched in the Scopus database from 2004 to 2024. After manual screening, a list of the 100 most-cited original articles was obtained, which was analysed for various factors, including temporal trends, subject areas, authorship patterns, country of origin, funding bodies, etc.

**Results:**

There has been a gradual decline over the past two decades in the number of publications. The United States was affiliated with the highest number of publications (n=61), followed by The United Kingdom (n=12) and Gambia (n=8). Several authors had 4 or more publications, with the highest number of publications by Rouse, B. T. (n=14). The American Journal of Ophthalmology takes the lead with 15 publications, while the National Eye Institute (n=23) was the leading funding body. Examination of institutional contributions shows that The University of Tennessee, Knoxville and UT College of Veterinary Medicine stand out with twelve publications each. Nearly half the publications belong to the field of medicine. However, significant publications also come from the fields Neuroscience, Microbiology and Immunology, Biochemistry, Genetics and Molecular Biology, etc. These findings highlight that there is great potential to conduct research to propagate ocular hygiene to prevent adverse effects of infections.

## Zusammenfassung

**Hintergrund:**

Die Augenhygiene umfasst ein Spektrum von Maßnahmen zur angemessenen Augenreinigung, zur Prävention von Augeninfektionen und zur Verhinderung von deren Ausbreitung. Die Infektionen betreffen alle Altersgruppen und können zu schweren Komplikationen wie Erblindung führen. Fast 1 Milliarde Fälle von über 2,2 Milliarden sehbehinderten Menschen weltweit hätten verhindert werden können. Die bibliometrische Analyse konzentriert sich auf Veröffentlichungen zum Thema Augenhygiene und Infektionen.

**Methode:**

Die Recherche wurde in der Scopus-Datenbank von 2004 bis 2024 durchgeführt. Nach manuellem Screening wurde eine Liste der 100 meist zitierten Originalartikel erstellt, die auf die Faktoren zeitliche Trends, Themenbereiche, Autorenschaft, Herkunftsland und Finanzierungseinrichtungen analysiert wurden.

**Ergebnisse:**

In den letzten zwei Jahrzehnten ist die Zahl der Veröffentlichungen allmählich zurückgegangen. Die meisten Veröffentlichungen stammen aus den Vereinigten Staaten (n=61), gefolgt vom Vereinigten Königreich (n=12) und Gambia (n=8). Mehrere Autoren hatten 4 oder mehr Veröffentlichungen, wobei Rouse (n=14) die meisten Veröffentlichungen hatte. Das American Journal of Ophthalmology steht mit 15 Veröffentlichungen an der Spitze. Das National Eye Institute (n=23) war mit der höchsten Anzahl von Veröffentlichungen beteiligt. Die Untersuchung der institutionellen Beiträge ergab, dass die Universität von Tennessee, Knoxville, und das UT College für Veterinärmedizin mit jeweils zwölf Veröffentlichungen hervorstechen. Fast die Hälfte der Veröffentlichungen entfällt auf den Bereich der Medizin. Aber auch in den Bereichen Neurowissenschaften, Mikrobiologie und Immunologie, Biochemie, Genetik und Molekularbiologie gibt es wichtige Veröffentlichungen. Die Ergebnisse verdeutlichen, dass es ein großes Forschungspotenzial zur Augenhygiene gibt, um die negativen Auswirkungen von Infektionen zu verhindern.

## Introduction

Ocular hygiene encompasses a spectrum of measures to initiate and maintain adequate ocular cleanliness to prevent eye infections and their further transmission. It plays a crucial role in reducing ocular morbidity and complications [1]. The most common eye infections associated with inadequate ocular hygiene include conjunctivitis, blepharitis, and orbital and peri-orbital cellulitis [2], [3]. These infections affect all age groups and are predominantly caused by Gram-positive bacteria such as *Staphylococcus aureus* and Streptococci, and Gram-negative bacteria such as *Pseudomonas aeruginosa* and *Haemophilus spp*. [4]. Their clinical course ranges from mild self-limiting eye manifestations, e.g., itching and inflammation, to severe complications such as blindness [5].

The World Health Organization estimates that over 2.2 billion people are visually impaired worldwide. Nearly 1 billion cases could have been prevented or can still be sufficiently managed. Among preventive measures, ocular hygiene remains a key reversible factor. Besides, optimal ocular hygiene is one of the most effective strategies to combat trachoma, a leading cause of permanent blindness [6].

In recent years, the number of research publications in the field of ophthalmology has markedly increased [7]. However, the volume and characteristics of publications on ocular hygiene and infections are yet to be determined. Bibliometric analysis is an important tool widely used to serve this purpose [8]. Previously, bibliometric analysis has been conducted in various areas such as dry eye, cataract surgery, myopia, orthopedics, and COVID-19 [9], [10], [11], [12], [13]. However, no similar bibliometric studies have been conducted on ocular hygiene and associated infections. 

We conducted the bibliometric analysis to quantitatively analyze the characteristics of the 100 most-cited studies on ocular hygiene and infections, and to assess the interconnectedness of key research themes to provide information for future directions in research and practice in the domain of ocular hygiene and related infections. We also intended to explore factors leading to an increase in the number of citations, such as publishing journal and publication time.

## Methods

The Scopus database was used to conduct the bibliometric analysis. Two reviewers were selected to search the database during November 2023. The first list comprised original articles published during 2004–2023. The original articles were separated from the review articles using Scopus filters. We included all the articles on Ocular hygiene and Ocular infection, using the following search term, (“ocular”[All Fields] OR “oculars”[All Fields]) AND (“hygiene”[MeSH Terms] OR “hygiene”[All Fields] OR “hygienic”[All Fields] OR “hygienical”[All Fields] OR “hygienically”[All Fields] OR “hygienics”[All Fields] OR “hygienization”[All Fields])) OR (“eye infections”[MeSH Terms] OR (“eye”[All Fields] AND (“infections”[All Fields]) OR “eye infections”[All Fields] OR (“ocular”[All Fields], AND (“infection”[All Fields]) OR “ocular infection”[All Fields]) OR ((“eye”[MeSH Terms] OR “eye”[All Fields]), AND (“hygiene”[MeSH Terms] OR “hygiene”[All Fields] OR “hygienic”[All Fields] OR “hygienical”[All Fields] OR “hygienically”[All Fields] OR “hygienics”[All Fields] OR “hygienization”[All Fields]). Review articles, guidelines, and those for which citation information was unavailable were excluded from the analysis.

The selected articles were related to ocular hygiene and ocular infection. The appropriateness and relevance were assessed by thoroughly going through the abstracts. In certain cases where abstracts were unavailable, we used other sources to obtain abstracts and determine their suitability using our inclusion criteria. The “cited by” filter in Scopus was used to arrange the articles in order of citations. We finally compiled the list of the 100 most-cited original articles. Only those articles were included to which both the reviewers agreed.

The analysis of the final list of articles was conducted using the Statistical Package for Social Sciences (SPSS) version 26 and Microsoft Excel, version 2016. The citation data includes total citations and citations per year. Microsoft Excel was used to visualize data through appropriate graphs and tables. Further, VOSviewer version 1.6.20 was used for visual analysis. Raw data from Scopus was uploaded in CSV format to retrieve the network visual analysis.

## Results

### Most cited publications

The top 100 most-cited publications are listed in Table 1 (Tab. 1). 

### Publication trends

**Figure 1:**
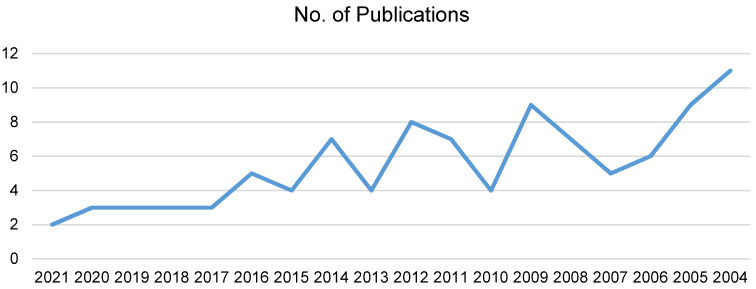
Trends in papers published per year

The temporal analysis of the 100 most-cited articles on ocular hygiene and ocular infection reveals intriguing patterns. There has been a decline over the past two decades in the number of publications. The average number of publications from 2004 to 2013 was 7 (Figure 1 (Fig. 1)).

### Citation analysis

**Figure 2:**
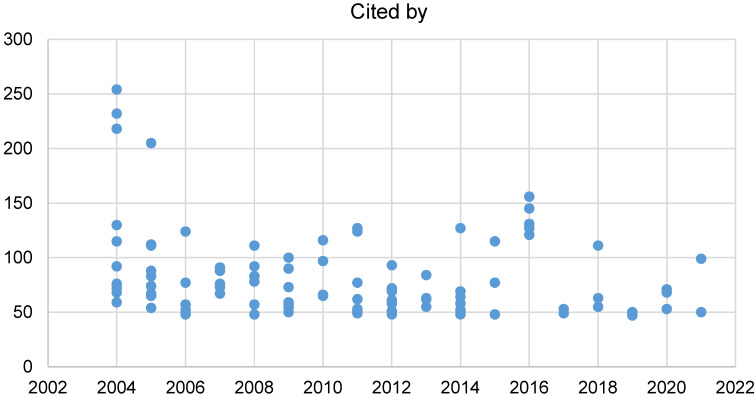
Scatter plot of the number of citations for each document (y-axis) vs. the year of publication (x-axis)

**Figure 3:**
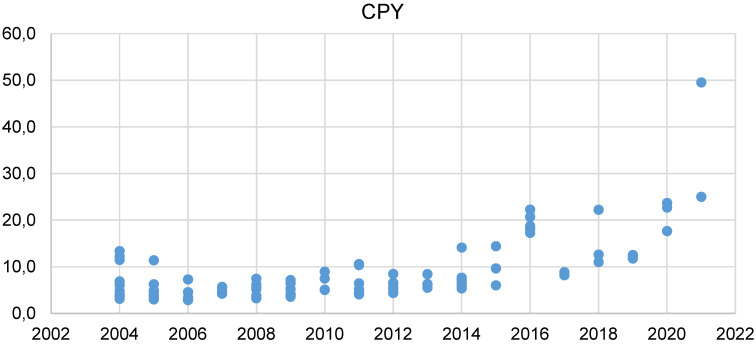
Scatter plot of the number of CPY of each document (y-axis) vs. the year of publication (x-axis)

Citations of each document versus their corresponding year of publication show a downward trend from 2004 to 2024 (Figure 2 (Fig. 2)). This is expected because the papers published in 2004 had a greater chance of being read and cited compared to the recent publications. Therefore, we also considered the factor of time since publication, and calculated citations per year (CPY) for a better analysis of trends. Hence, Figure 3 (Fig. 3) shows a completely opposite trend. It shows that the CPY has increased from 2012 to 2023, with the study “COVID-19 retinal microangiopathy as an in vivo biomarker of systemic vascular disease?” having the highest CPY of 50. 

### Distribution across source titles

The distribution of publications across source titles underscores the preferred avenues for disseminating ocular hygiene and ocular infection research. The American Journal of Ophthalmology takes the lead with 15 publications, followed by Ophthalmology (8) and Retina (6) (Table 2 (Tab. 2)). 

### Authorship patterns

Analyzing authorship patterns reveals key contributors and collaboration dynamics. Prolific authors, including Rouse BT, Bailey RL, and Holland MJ, emerged as influential figures with more than seven publications each (Table 3 (Tab. 3)). The collaborative spirit is evident, with several authors contributing more than three publications, indicating a network of researchers pooling expertise. This collaborative approach fosters a comprehensive exploration of ocular hygiene and ocular infection from various perspectives.

### Affiliation analysis

**Figure 4:**
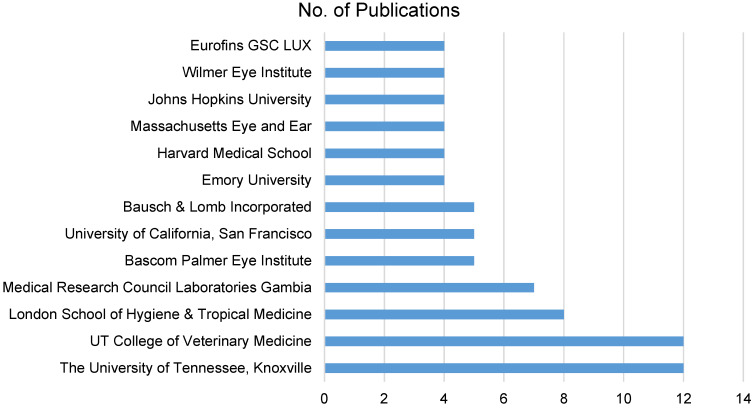
Institutions with more than 3 publications

An in-depth examination of affiliations highlights institutional contributions. The University of Tennessee, Knoxville, TN, USA and UT College of Veterinary Medicine (also Knoxville) stand out with 12 publications each, indicating their centrality in ocular hygiene and ocular infection research (Figure 4 (Fig. 4)). This collaborative effort extends globally, involving institutions in the United States, Gambia, India, and Italy. The diversity in affiliations enriches the research landscape, offering varied insights into the condition.

### Country distribution

**Figure 5:**
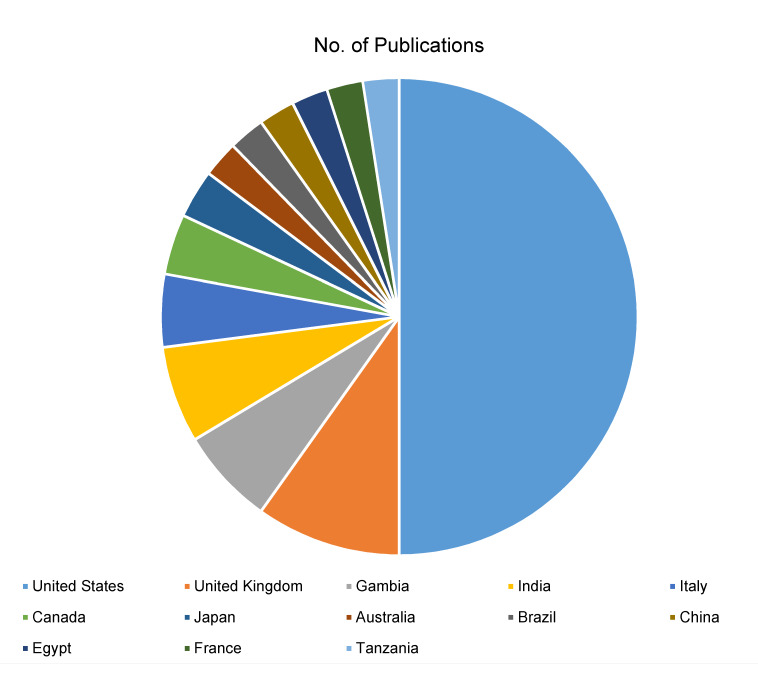
Country-wise distribution

The geographical distribution of publications demonstrates the global footprint of ocular hygiene and ocular infection research. The United States leads with 61 publications, followed by United Kingdom (12) and Gambia (8) (Figure 5 (Fig. 5)). This international collaboration enriches the field by incorporating diverse perspectives and approaches. The global nature of research not only broadens the knowledge base but also facilitates the development of universally applicable insights and interventions.

### Subject area distribution

**Figure 6:**
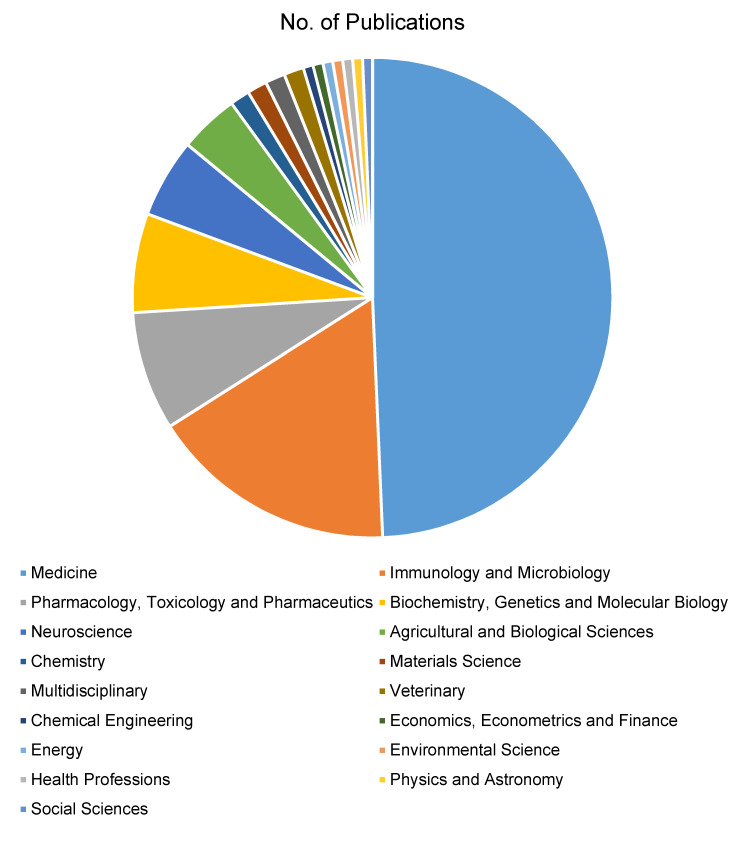
Subject-wise distribution xxre-think “medicine”, see comments above

A meticulous examination of subject areas reveals the interdisciplinary nature of ocular hygiene and ocular infection research. Most publications fall within the medicine subject area, emphasizing their clinical relevance. Significant contributions in neuroscience and nursing highlight the broader impact and collaborative nature of the research. This interdisciplinary engagement reflects a holistic approach toward understanding and addressing ocular hygiene and ocular infection. Further close collaboration from departments of Immunology and Pharmacology was not only evident but also predictable given the pivotal role of Immunology and Pharmacology in the development and resolution of ocular infections (Figure 6 (Fig. 6)).

### Funding organizations

**Figure 7:**
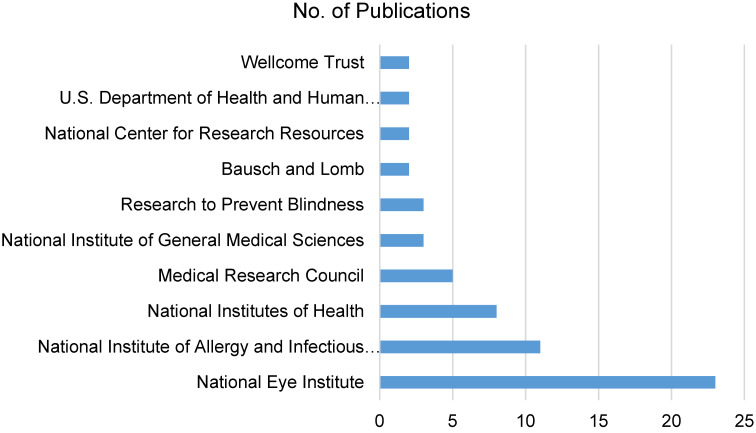
Funding institutions with more than 1 publication

The financial landscape supporting ocular hygiene and ocular infection research is crucial. Noteworthy organizations such as the National Eye Institute, National Institute of Allergy and Infectious Diseases, and National Institutes of Health lead with 23, 11, and 8 publications, respectively. Diverse funding sources, both national and international, emphasize the broad support for research in this field (Figure 7 (Fig. 7)). 

### Keyword analysis

**Figure 8:**
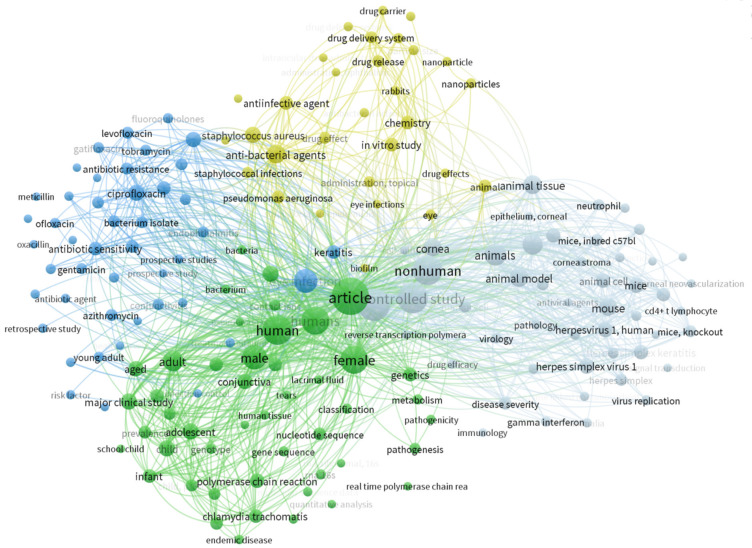
Index Keyword analysis

The keyword analysis was conducted on the author-indexed keywords through the “VOS viewer” software, resulting in a total of 171 keywords scattered among 4 dense clusters. The cluster shown in blue color in Figure 8 (Fig. 8) shows relatively closer associations of antimicrobials to each other as well as their corresponding correlation with their causative agents in the yellow cluster. Similarly, gray and green clusters show their association with in-vivo studies on mice and studies on humans, respectively. 

### Collaboration networks

**Figure 9:**
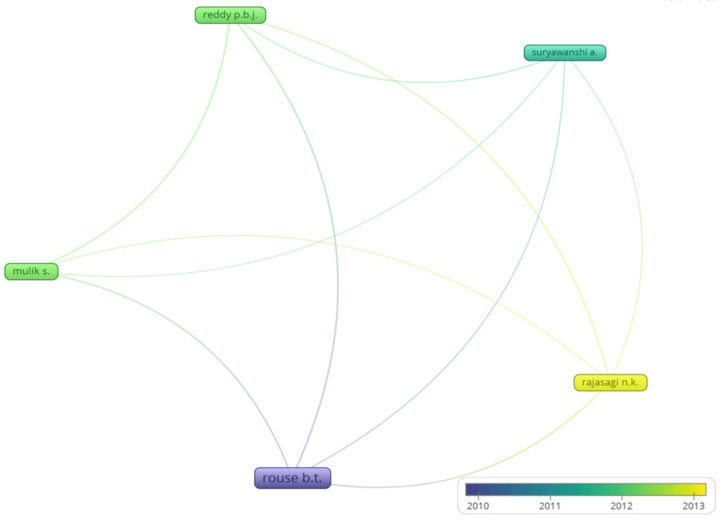
Co-authorship Analysis

The settings for VOS viewer for co-authorship network analysis were kept as minimum documents=4. Therefore, the qualifying authors were 12. Among them, only 5 had collaborations, as shown in Figure 9 (Fig. 9). 

## Discussion

This study aimed to provide a thorough evaluation of the most-cited articles on ocular hygiene and infections, to provide possible research hot spots and offer vital data for unexplored and future research areas in this field that will be useful to researchers and physicians. It can also promote cooperation between academics from many disciplines, enabling them to collaborate and delve further into topics related to ocular infection and hygiene research.

We have observed a declining trend in research productivity in this field, with the greatest number of articles in 2004 (i.e., xx?) and the least number in 2021 (i.e., two). This contrasts with the other areas of ophthalmology, such as dry-eye disease, refractive cataract surgery, myopia [9], [10], and diabetic retinopathy [14], where bibliometric analyses have shown an increasing research yield over the years. Priorities and areas of interest for medical research might shift over time. Resources for ocular infections may be diverted by newly discovered illnesses, infections, or ailments that receive greater attention. For example, the COVID-19 pandemic caused a significant shift in the resources and focus of study [15]. Another possible reason for this can be the decrease in the percentage of blindness caused by ocular infections after the introduction of antibiotics [16]. 

Nevertheless, the prevalence of ocular infections in developing countries is still a burden to their already compromised healthcare systems. WHO data from June 2022 indicate that 125 million individuals are still at risk of trachoma blindness; they reside mainly in the endemic locations for the disease. These are the poorest and most rural areas of Africa, Central and South America, Asia, Australia, and the Middle East [6]. Thus, it is imperative that research on ocular flora and hygiene continue, since known infections are always adapting and new pathogens are constantly emerging. 

Citation count was found to be highest for the study “The severe acute respiratory syndrome coronavirus in tears”. Literature suggests that citation count alone is not a reliable enough indicator of an article’s scientific merit. This is because of the fact that fewer recent publications are included in the top 100 list of articles, as they are less frequently cited in their initial years of release, and also because the generation time is shorter for their citation frequencies [17]. Hence, we also selected citations per year (CPY) as a more reliable metric to assess an article’s current effect. A relatively recent article by Landecho et al. [18] was found to have the highest CPY of 50. This high CPY suggests that the recent research is of high quality and includes insightful observations, creative solutions, or significant discoveries that have a significant impact on the field of ophthalmology. It was found that a total of 11 authors contributed to 4 or more articles in the list. The collaborative spirit is evident, indicating a network of researchers pooling expertise. This collaborative approach fosters a comprehensive exploration of ocular hygiene and ocular infection from various perspectives. However, there was only one female among the top-performing authors. This data corroborates the findings of previously conducted studies that demonstrate the under-representation of female authors in ophthalmology [7], [10] and other medical specialties [13], [19], [20], [21]. A lower number of female ophthalmology residents and specialists as compared to their male counterparts explains this finding. Moreover, gender bias in granting funds for research also contributes to the low research productivity of females [22]. This indicates that more work needs to be done to support women’s inclusion and participation in the workforce and in leadership positions in ophthalmology. 

The analysis of countries of origin showed that The United States led with 61 publications, followed by The United Kingdom (n=12) and Gambia (n=8). Moreover, the top institutes with a maximum number of research articles were also from the US and the UK. Previous bibliometric analyses done in ophthalmology and other fields of medicine also support this trend [7], [10], [12], [23], [24], [25]. This enormous contribution of the United States to ocular infection-related research can be attributed to its large-scale government and private sector funding to ophthalmology-related research, a greater number of high-source journals, labs equipped with advanced equipment, a robust higher education system, and elaborate collaborative networks [26]. Moreover, the top-performing researchers were also from the US. The United Kingdom also has a robust research infrastructure, being third in the world with respect to scientific output. While ocular infections are more prevalent in the developing countries of Africa and Asia [6], research output from this region is still lagging, with only Gambia and India making significant contributions. This points to a dire need for continual exploration of novel aspects in the area of ocular hygiene and infections, possibly fueled by technological advancements and emerging therapeutic interventions with a focus on the epidemiological factors in the endemic countries.

Most of the articles in this bibliometric analysis received funding. The funding bodies that contributed to the maximum number of articles, i.e., the National Eye Institute, National Institute of Allergy and Infectious Diseases, and National Institutes of Health, were all from the US. This robust financial backing has played a pivotal role in the US lead in advancing investigations and translating its findings into clinical practice. Not even a single funding body was found to be based in the endemic developing countries. Although the US and other foreign funding bodies invest in research and xx? development in low-income countries, it is irregular, insufficient, and comes with strict requirements. The result of this is that despite lower labor costs, researchers in the developing world face a double funding crisis: lower absolute levels of funding than in the West and greater research expenditures [27]. As a result, it emphasizes how crucial it is to create a comprehensive local-health finance strategy for these nations, designating at least 15% of the national budget for health development [28], [29].

Top source journals also came from the US with the Journal Of Immunology and the American Journal of Ophthalmology contributing to a maximum number of articles. Beyond the ophthalmology realm, the inclusion of journals such as the Journal of Virology, American Journal of Pathology, Antimicrobial Agents and Chemotherapy, and Genomic Medicine reflects the interdisciplinary nature of the research. This diverse dissemination strategy contributes to a holistic understanding of ocular hygiene and ocular infection. This observation also suggests that authors and researchers from a wide range of disciplines are interested in ocular hygiene and infection, and that research in this area is disseminated to a diverse readership within the domains of microbiology, pathology, pharmacology, epidemiology, and genetics.

Keyword analysis showed that the most common themes in this subject area were antimicrobial agents and infection-causing microbes based on human and mouse studies. Antimicrobial sensitivity, antibiotic resistance, the role of immune mediators and T cells in the pathogenesis of ocular infections, normal ocular microbiota, the role of genetic diversity among different bacterial strains in ocular infections, potent drug delivery systems and molecular biology of ocular infections for developing effective targeted drugs were the trending spots. The scrutiny of keywords presents an illuminating panorama of prevalent themes and nuanced topics within the dynamic field of ocular hygiene and ocular infection research. Unearthing frequently employed keywords provides a panoramic snapshot of pivotal focus areas and the evolutionary trajectory of research trends.

There are certain limitations to our results. First, since only the Scopus database was used, some papers may have been overlooked. Scopus often ignores articles published before the 2000s, when an automated method was implemented [30]. Second, self-citations remained in the text. Thirdly, a bias in the publishing language selection process may arise from most of the publications being published in English.

## Conclusions

Our analysis has provided an insight into the most influential articles published in the field of ocular hygiene and infections in the last two decades. There has been a gradual decline in the number of publications. The United States showed the greatest research productivity and the highest number of funding bodies. The most common themes in this subject area were antimicrobial agents and infection-causing microbes based on human and mouse studies. Still, the high prevalence of ocular infections in the endemic developing nations highlights the need for international collaboration between authors and increased funding.

## Notes

### Autho’s ORCID-ID

- Mohsan Ali is:0000-0002-5697-0458.
- Rawal Alias Insaf Ahmed is: 0009-0009-7033-2354.

### Competing interests

The authors declare that they have no competing interests.

## Figures and Tables

**Table 1:**
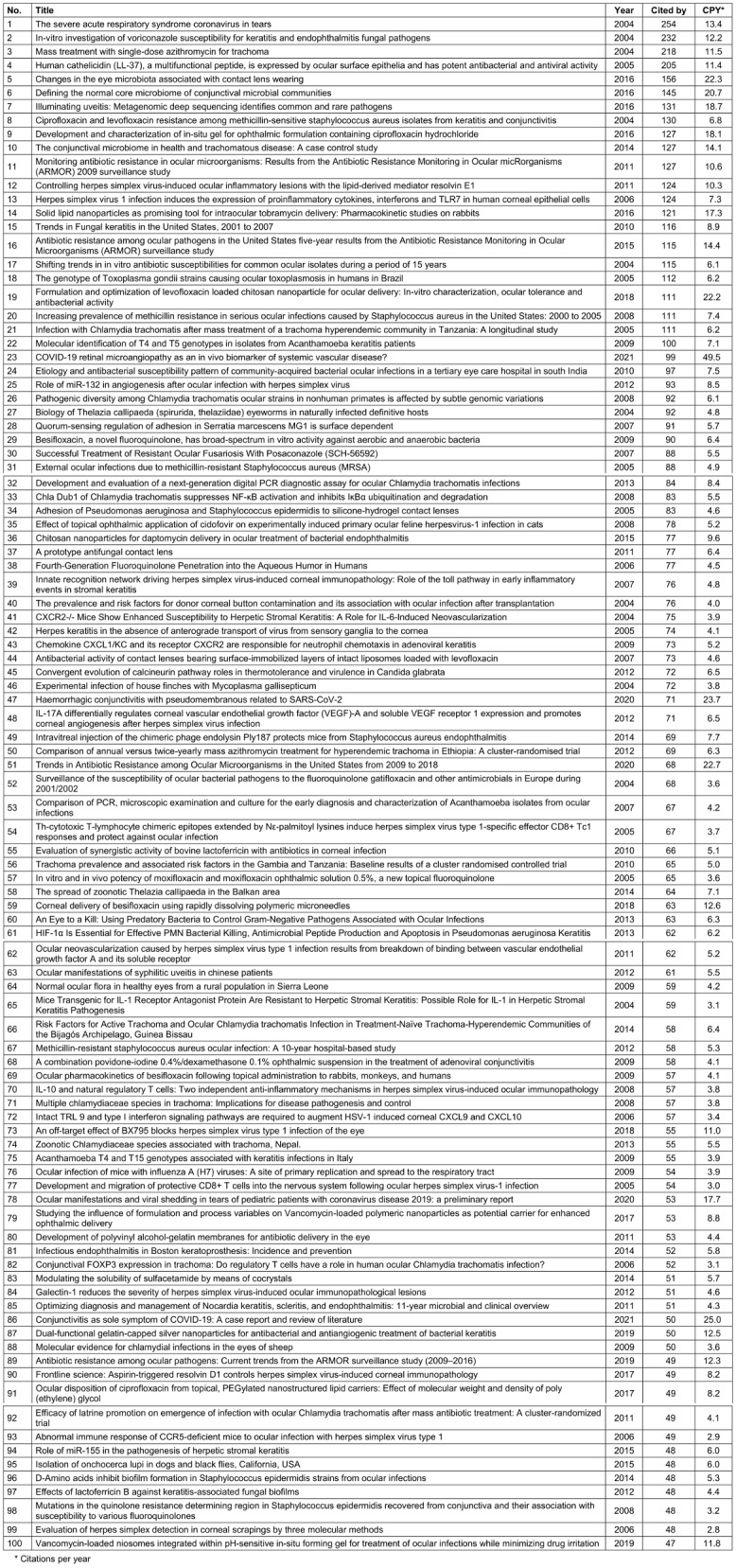
List of the top 100 most-cited publications

**Table 2:**
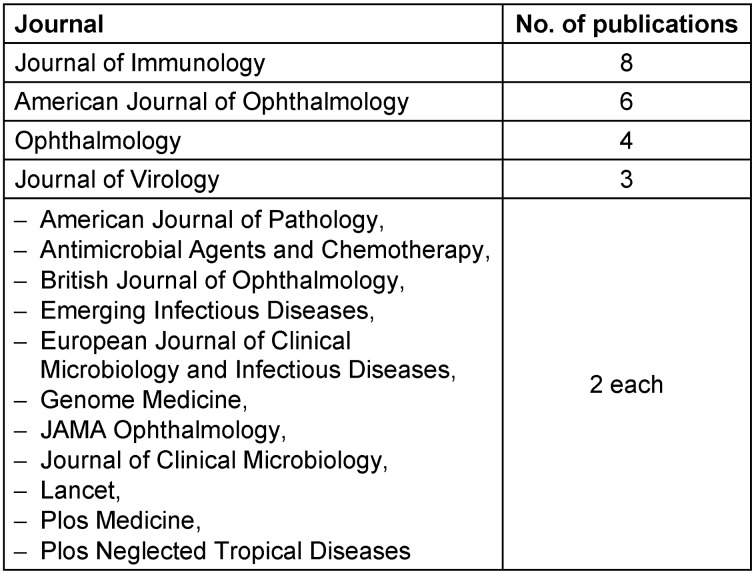
Journals with more than 1 publication

**Table 3:**
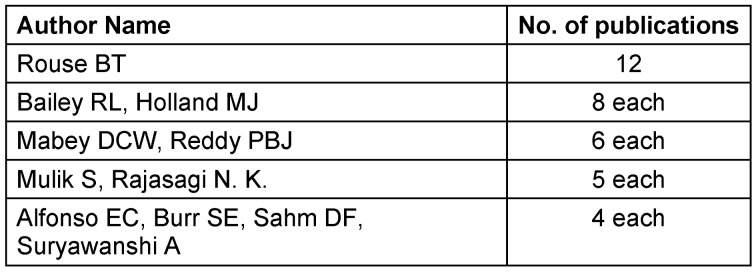
Authors with more than 3 publications

## References

[1] Alhamazani MA, Alnabri MS, Alreshidi MN, Alsulaiman HM, Strianese D, Althaqib RN (2021). Assessing public awareness of daily eyelid hygiene habits in Saudi Arabia: An online survey study. Saudi J Ophthalmol.

[2] Sharma S (2011). Antibiotic resistance in ocular bacterial pathogens. Indian J Med Microbiol.

[3] Falavarjani KG, Nekoozadeh S, Modarres M, Parvaresh MM, Hashemi M, Soodi R, Alemzadeh SA (2012). Isolates and antibiotic resistance of culture-proven endophthalmitis cases presented to a referral center in Tehran. Middle East Afr J Ophthalmol.

[4] Castañeda-Sánchez JI, García-Pérez BE, Muñoz-Duarte AR, Baltierra-Uribe SL, Mejia-López H, López-López C, Bautista-De Lucio VM, Robles-Contreras A, Luna-Herrera J (2013). Defensin production by human limbo-corneal fibroblasts infected with mycobacteria. Pathogens.

[5] Chaudhary M, Bhattarai A, Adhikari SK, Bhatta DR (2010). Bacteriology and antimicrobial susceptibility of adult chronic dacryocystitis. Nepal J Ophthalmol.

[6] World Health Organization (2022). Fact Sheet Trachoma.

[7] Boudry C, Baudouin C, Mouriaux F (2018). International publication trends in dry eye disease research: A bibliometric analysis. Ocul Surf.

[8] Mejia C, Wu M, Zhang Y, Kajikawa Y (2021). Exploring Topics in Bibliometric Research Through Citation Networks and Semantic Analysis. Front Res Metr Anal.

[9] Wu Q, Xie M, Li S, Li S, Tian L, Jie Y (2023). Mapping the research on Sjögren's syndrome-related dry eye disease: a bibliometric network analysis of the past 20 years. Int Ophthalmol.

[10] Chen XY, Wu QR, Xie MY, Zhang D, Zhang C (2023). Bibliometric analysis of research relating to refractive cataract surgery over a 20-year period: from 2003 to 2022. Int J Ophthalmol.

[11] Shan M, Dong Y, Chen J, Su Q, Wan Y (2022). Global Tendency and Frontiers of Research on Myopia From 1900 to 2020: A Bibliometrics Analysis. Front Public Health.

[12] Li C, Foster AL, Han NHB, Trampuz A, Schuetz M (2022). A bibliometric analysis of clinical research on fracture-related infection. Biomed Res Int.

[13] Naseer B, Ali M, Azhar N (2023). COVID-19 research in South Asia: a bibliometric analysis of the 100 most-cited articles. GMS Hyg Infect Control.

[14] Xiao H, Tang J, Zhang F, Liu L, Zhou J, Chen M, Li M, Wu X, Nie Y, Duan J (2023). Global trends and performances in diabetic retinopathy studies: A bibliometric analysis. Front Public Health.

[15] Funada S, Yoshioka T, Luo Y, Iwama T, Mori C, Yamada N, Yoshida H, Katanoda K, Furukawa TA (2023). Global Trends in Highly Cited Studies in COVID-19 Research. JAMA Netw Open.

[16] Wilhelmu KR (2016). Epidemiology of Ocular Infections.

[17] Harzing AW (2010). Basic metrics based on papers and citations.

[18] Landecho MF, Yuste JR, Gándara E, Sunsundegui P, Quiroga J, Alcaide AB, García-Layana A (2021). COVID-19 retinal microangiopathy as an in vivo biomarker of systemic vascular disease?. J Intern Med.

[19] Sheikh MH, Chaudhary AMD, Khan AS, Tahir MA, Yahya HA, Naveed S, Khosa F (2018). Influences for Gender Disparity in Academic Psychiatry in the United States. Cureus.

[20] Tanvir I, Hassan A, Alahmadi S, Waseem H, Anwer J, Shafie A, Sheikh MA, Elbasateeny SS, Khosa F (2023). Ethnic and Gender Diversity in Pathology: A Dream Deferred. Cureus.

[21] Merone L, Tsey K, Russell D, Nagle C (2022). Sex Inequalities in Medical Research: A Systematic Scoping Review of the Literature. Womens Health Rep (New Rochelle).

[22] Morgan R, Hawkins K, Lundine J (2018). The foundation and consequences of gender bias in grant peer review processes. CMAJ.

[23] Sun HL, Bai W, Li XH, Huang H, Cui XL, Cheung T, Su ZH, Yuan Z, Ng CH, Xiang YT (2022). Schizophrenia and Inflammation Research: A Bibliometric Analysis. Front Immunol.

[24] Ahmad T, Murad MA, Baig M, Hui J (2021). Research trends in COVID-19 vaccine: a bibliometric analysis. Hum Vaccin Immunother.

[25] Zhang C, Luo M, Zhu H, Zhou J, Miao L (2022). The 100 Most-Cited Articles in the Field of Colorectal Diseases from 1955 to 2020: A Bibliometric Analysis. Turk J Gastroenterol.

[26] National Center for Science and Engineering Statistics (NCSES) (2021). National Patterns of R&D Resources: 2018–19 Data Update. NSF 21-325.

[27] van Helden P (2012). The cost of research in developing countries. EMBO Rep.

[28] Kirigia JM, Barry SP (2008). Health challenges in Africa and the way forward. Int Arch Med.

[29] Khayyat NT, Lee JD (2015). A measure of technological capabilities for developing countries. Technological Forecasting and Social Change.

[30] Falagas ME, Pitsouni EI, Malietzis GA, Pappas G (2008). Comparison of PubMed, Scopus, Web of Science, and Google Scholar: strengths and weaknesses. FASEB J.

[31] AlRyalat SAS, Malkawi LW, Momani SM (2019). Comparing Bibliometric Analysis Using PubMed, Scopus, and Web of Science Databases. J Vis Exp.

[32] Pranckutė R (2021). Web of Science (WoS) and Scopus: The titans of bibliographic information in today's academic world. Publications.

